# Modifying the Properties of Construction Mixtures Containing Crushed Concrete Waste for 3D Printing

**DOI:** 10.3390/ma19050877

**Published:** 2026-02-26

**Authors:** Vitaliy Marchuk, Ruslan Makarenko, Leonid Dvorkin, Yuri Ribakov

**Affiliations:** 1 Institute of Civil Engineering and Architecture, National University of Water and Environmental Engineering, 33028 Rivne, Ukraine; v.v.marchuk@nuwm.edu.ua (V.M.); r.m.makarenko@nuwm.edu.ua (R.M.); l.i.dvorkin@nuwm.edu.ua (L.D.); 2 Department of Civil Engineering, Ariel University, Ariel 40700, Israel

**Keywords:** 3D printing, cement pastes, complex additive, setting time, normal consistency, regression equations, viscosity, plastic strength, cement–sand mortars, water separation, compressive strength, flexural strength

## Abstract

The paper presents the results of a study focused on technological parameters that ensure the effectiveness of using concrete powders obtained from crushed concrete waste as an active mineral additive in construction mixtures used for 3D printing. The efficiency of using a complex polyfunctional additive to cement pastes and mortars based on it is experimentally demonstrated. This additive includes a naphthalene–formaldehyde-based superplasticizer and a water-retaining additive, hydroxyethyl methylcellulose. Using the mathematical experiment planning methodology, polynomial models of the cement pastes and mortars’ mechanical properties were obtained. The models showed a positive effect of the complex additive on the cement pastes’ normal consistency and viscosity. Additionally, the results of the study demonstrate the possibility of regulating the cement pastes’ setting time and the plastic strength of mortars based on them using a complex additive. Analysis of the experimental–statistical models shows that using the complex additive allows regulation of water separation as well as the compressive and flexural strength of cement–sand mortars based on the investigated cement pastes within the required limits. Improving the key properties of building mixtures containing crushed concrete waste for 3D printing using complex polyfunctional modifier additives opens up new opportunities for increasing their economic and environmental efficiency.

## 1. Introduction

3D printing is increasingly used in construction to accelerate building processes, reduce labor costs, and enable complex architectural designs. Increasing the economic and environmental performance of buildings using 3D printing makes it advisable to use technological mortars aimed at reducing Portland cement consumption. This is especially relevant considering that 3D printing technology mainly uses fine-grained concrete mixtures and cement–sand mortars. Lowering cement use also reduces the carbon footprint, typically through mineral additives with pozzolanic activity, such as natural rocks and industrial by-products. Common additives include blast furnace slag and coal ash; however, high demand has led to shortages in some regions, including Ukraine.

The dismantling, repair, and reconstruction of old buildings, along with damage caused by military actions in Ukraine, generate large amounts of construction waste, primarily concrete scrap (CS). In 2018, European Union countries produced 374 million tons of construction waste, and this volume continues to grow annually [1,2,3].

Studies show that concrete powder (CP) derived from CS can function as an active mineral additive [4,5,6]. The action mechanism of such additives in concrete and mortar has been investigated [7,8,9]. At the same time, the concrete’s composition and structure determine the peculiarities of this mechanism and its influence on hardening.

At the microlevel, the activity of mineral additives is manifested in the results of chemical and physicochemical processes. Chemical processes, when introducing recycled CPs, are manifested through the hydration of cement grains that have not yet reacted with water. In addition, the appearance of CP pozzolanic activity is possible due to the composition and amorphized surface of mainly hydrated compounds.

Following the Gibbs–Volmer theory [10,11,12], the physicochemical activity of CPs is due to the development of crystal nuclei of new formations of hardening concrete and mortar, playing the role of crystallization centers [13,14].

Chemical and physicochemical activation of CPs should increase with increasing their specific surface area, which is accompanied by an increase in surface energy and in isobaric potential. Also, reducing the intergranular distances allows for increasing the strength of the contact zone between the binder and aggregates, which has a positive effect on both compressive and flexural strength, as well as on adhesive strength [9,15,16,17,18,19,20,21].

The effectiveness of adding CP into concrete and mortar can be estimated by the ratio of the 28-day compressive strength of concretes and mortars with CP to the corresponding strength without filler. Without additional activation methods, the activity index of concrete and mortar with a CP content of 20 ... 30% usually does not exceed 60 ... 70% [22].

Promising methods for activating CP are grinding to a higher specific surface area [9,20,23,24], heat treatment [9,25] and using chemical additives that reduce the water demand and accelerate hardening [9,26,27,28]. The effectiveness of activation methods lies in increasing the hydration rate of cement with filler, improving the microstructure and reducing porosity.

The main studies of CP as fillers have been carried out for ordinary concrete and general-purpose mortars [29,30,31,32,33]. Analysis of available results shows that superplasticizer additives, while liquefying concrete mixtures and reducing their water consumption, do not provide sufficient viscosity or the required plastic strength during structural molding [14,22].

Construction mixtures for 3D printing should have a number of special properties, in particular the required extrudability, depending on their viscosity and plasticity. A characteristic feature of such mortar mixtures is their increased water demand, which can be reduced by adding superplasticizers (SP). However, in this case, there is a need to regulate the viscosity to ensure the required mixture extrudability, which makes it advisable, along with the use of superplasticizers, to introduce additional chemical additives that allow maintaining the required viscosity and water-holding capacity. In this case, it is necessary to ensure a sufficient “printing window”, which depends on the setting time and the kinetics of plastic strength growth.

Additive technologies require that the previous layers achieve the required structural and adhesive strength before laying subsequent layers of concrete or mortar, which makes it advisable to use in the mixtures appropriate complex additives, which act as polyfunctional modifiers (PFM).

For structures formed using additive technology, the problem of ensuring sufficient crack resistance is particularly relevant. The general theoretical foundations of the crack resistance of concrete have been developed in many works [7,34,35].

A strategy of phases for modeling the appearance of cracks and their propagation in heterogeneous materials, including materials obtained by 3D printing, was proposed [35]. The proposed models allow predicting the conditions of dynamic crack development and their prevention, as well as developing optimal building structures produced using additive technology.

Crack resistance in 3D-printed concrete is established during manufacturing and must ensure sufficient structural and interlayer adhesive strength to provide bearing capacity and durability. These properties depend on the concrete composition. Since CP is compositionally similar to the original concrete, it may enhance crack resistance. However, its higher porosity and water demand require careful adjustment of the mix design when used as an active filler.

Despite growing interest in using CP as an active mineral additive and the increasing application of 3D printing in construction, there is insufficient understanding of how CP interacts with complex chemical admixtures in cement-based systems designed for additive manufacturing. In particular, the combined influence of CP content and multicomponent chemical additives on the rheological, structural, and strength properties of cement pastes and cement–sand mortars for 3D printing requires further investigation. Moreover, there is a lack of quantitative predictive models that describe how variations in CP dosage and admixture composition affect the characteristics of construction mixtures for 3D-printing. Addressing this gap is essential for proper mix design, ensuring printability, interlayer adhesion, and mechanical performance while reducing Portland cement consumption.

## 2. Materials and Methods

Experimental studies were performed using:

- Portland cement CEM I 42.5 R (EN 197-1). The mineralogical and chemical composition of the used Portland cement clinker according to EN 196-2 is given in Table 1 and Table 2. The specific surface area of Portland cement was S_CEM_ = 330–350 m^2^/kg according to the Blaine Air method (BS EN 196-6; 2018).
- Concrete powder obtained from concrete scrap of concrete class C16/20. It had a specific surface area S = 160 … 180 m^2^/kg (BS EN 196-6; 2018) and residues on sieves 45, 63, 90 µm: Q_45_ = 35.37 … 37.32%, Q_63_ = 39.41 … 41.53%, Q_90_ = 41.53 … 48.47%, Q_125_ = 0.0% (EN 933-1), consistency—2.9 g/cm^3^, bulk consistency—1280 kg/m^3^ (EN 1097-6). The mineralogical and chemical composition of CP determined by EN 196-2 and EN 932-3 is given in Table 1 and Table 2.
- Naphthalene formaldehyde type superplasticizer—SNF–C (EN 934-2), with a water-reducing effect of 20%.
- Additive to increase the viscosity and water-holding capacity of mixtures—a modified hydroxyethyl—methylcellulose (ethers of cellulose—EC). Viscosity of 2% solution in water, Haake Rotovisko RV 100 (Thermo Fisher Scientific, Waltham, MA, USA), shear rate 2.55 s^−1^, 20 °C—20,000 mPa s, solution pH—8, solution stability without sediment—1 … 2 weeks.
- Quartz sand (EN 933-1, EN 933-8) with a fineness modulus of 2.1, and a content of dusty and clay particles up to 1.0%.

Considering that the rheological properties of mortar mixtures are determined mainly by the properties of cement pastes, on the basis of which the mixtures were made, the research was carried out in two stages: (1) cement pastes with complex additives; (2) cement–sand mixtures. Normal consistency (NC) and the kinetics of hardening of the pastes were determined on a Vicat device.

To determine the viscosity, a rotational viscometer RV-8M (AMETEK Brookfield, Middleboro, MA, USA) was used. An important advantage of rotational rheometry is the possibility of implementing practically unlimited shear deformations with a given speed. The RV-8M device belongs to rotational hemispherical–cylindrical viscometers; its main feature is the ability to consider bottom and end effects. Pastes with W/C = 0.3 were studied when determining rheological properties for 5 min. after mixing. The paste viscosity was obtained using the following equation:

(1) $$\eta =k\frac{P-P_{o}}{N}\tau ,$$

where *k* is the device constant; P—the cargo weight; P_o_—the device idling load (P_o_ = 12.4 g); N—the number of revolutions; *τ—*revolution time. The cylinder rotation frequency varied from 27 to 430 s^−1^.

**Figure 1:**
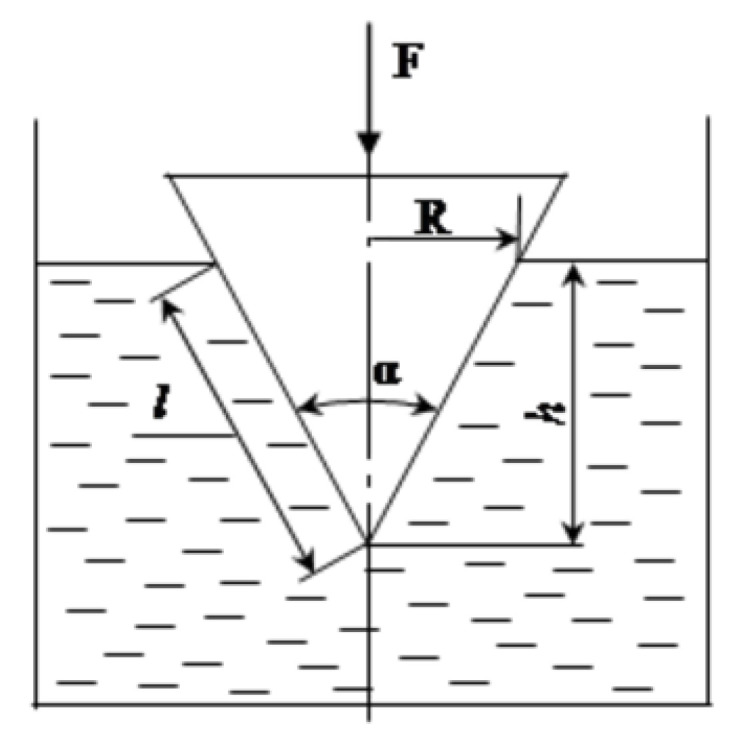
Scheme of plastic strength testing by the conical plastometer method.

The plastic strength value in the cement pastes’ initial setting time was obtained by a conical plastometer (Figure 1).

The cone immersion depth was recorded by an indicator with a division value of 0.01 mm. Calculations were performed according to the following equation:

P*_m_* = 0.096 F/*h*^2^, (2)

where F is the weight of the cone with the load, N; *h* is the cone immersion depth, mm.

To determine the water separation, the mortar mixtures were prepared at constant plasticity, which was determined according to EN 196-1 on a shaking table. Required plasticity is necessary to ensure the mixtures’ extrudability from the printer nozzle. According to DSTU B V.2.7-186 (Ukrainian standard), the mortar mixtures’ volumetric water separation coefficient (K*_v_*, %) was obtained:

(3) $$K_{v}=\frac{a-b}{a}\cdot 100\%,$$

where *a* is the initial volume of cement suspension, cm^3^ and *b* is the volume of cement suspension that has settled, cm^3^.

The experiments were performed with two repetitions. The mortar mixtures’ workability was obtained by the standard cone immersion, flexural, splitting tensile and compressive strength at 28 days according to EN 196-1.

The “formability window” was estimated by the initial setting of the mixtures following EN 196-3, using the time between the moment of mixing and the initial setting, when further molding using a 3D printer is impossible.

Methods of mathematical planning of experiments were used to conduct the research. With this aim, algorithmized experiments were carried out following the three-level three-factor B_3_ plan [36].

The mathematical modeling task was to build experimental and statistical models, which have the following general form:

(4) $$y=b_{0}+\sum_{i=1}^{k}b_{i}x_{i}+\sum_{i=1}^{k}b_{ij}x_{i}x_{j}+\sum_{i=1}^{k}b_{ii}x_{1}^{2},$$

where *y* is the optimization parameter, i.e., the investigated parameter of the system, *b_i_*, *b_ii_*, *b_ij_* are regression coefficients; *x_i_*, *x_j_* are factors; *k* is the number of factors.

Quadratic-type regression equations allow tracing the factors’ individual and joint influence on the investigated initial parameters, to establish the necessary optimal factors’ values.

## 3. Results and Discussion

### 3.1. Rheological and Structural–Mechanical Properties of Cement Pastes with Addition of CP

As is known, water demand and water separation of mortar mixtures are determined by the cement’s normal consistency [9], which is linearly related to the maximum molecular moisture capacity and ultimate water-holding capacity of cement paste and depends on the mineralogical composition, dispersion of cements, and the amount and type of additives. At the initial research stage, the influence of the complex polyfunctional modifier additives (PFM) components content and the CP/C ratio on the cement pastes’ normal consistency was determined. The results given in Table 3 indicate that using CP increases the normal consistency (NC) of the composite binder based on CP. Addition of SP reduces the NC of cement pastes, both without CP and with its content up to 30% by the cement weight. However, in mixtures containing SP and EC, the NC practically does not decrease, which in turn allows for ensuring low water separation [9] and sufficient viscosity of mortar mixtures. Obviously, in this case, the effect of reducing the water requirement and, accordingly, the normal density of cement pastes with SP additives is balanced by the effect of increasing the normal density due to the EC additive.

Further studies were carried out to obtain quantitative relationships, allowing for predicting the pastes’ NC values and effective viscosity, depending on the amount of CP and complex modifier. The studies were performed following a typical three-level B_3_ plan [34]. Algorithmic experiments were performed using SP and EC polyfunctional modifier additives, Portland cement and concrete powder. The planning conditions of the experiments are given in Table 4. At the same time, in the experiment planning matrix (Table 5), there are points where the studied pastes do not contain one or both additives.

To prepare cement pastes in accordance with the planning conditions at certain points of the plan, 1 kg of cement was first prepared and mixed in a laboratory paddle mixer with the required amount of CP. Then, the water-retaining additive EC was dissolved in water in the amount specified in the plan. An SP additive was added to the resulting binder, consisting of a mixture of cement and CP, with additional mixing in accordance with Table 4. The resulting plasticized binder was mixed with an aqueous solution containing EC until the paste flowed on a Suttard viscometer of 200–220 mm. On the pastes specified in the experimental planning conditions (Table 4), 5 min after mixing, the normal consistency was checked using a Vicat apparatus, and the viscosity was checked with a ratio viscometer. The planning matrix in Table 5 shows the average values of the specified parameters from three measurements at each point of the matrix. Constant workability of 200 … 220 mm was ensured at all plan points.

The regression equations of normal consistency and effective viscosity (*η_ef_*) for cement pastes with CP were obtained by statistical processing. The obtained results are presented in Table 6. Statistical analysis showed their adequacy at a 95% confidence level. The obtained equations can be considered as mathematical models of the cement pastes’ properties in the selected factor space [36].

It can be noted that the obtained experimental and statistical models with different sets of factor values describe changes in the normal consistency and viscosity of both cement pastes without CP (X_3_ = −1), with CP (X_3_ > −1), with the modifying additives (X_1_ > −1, X_2_ > −1) and without them (X_1_ = −1, X_2_ = −1). At the same time, the variation range of factors X_1_ and X_2_ allows us to trace the influence on the investigated properties for a wide range of PFM compositions, including extreme compositions, when PFM is represented by only one component (superplasticizer or water-retaining additive).

**Figure 2:**
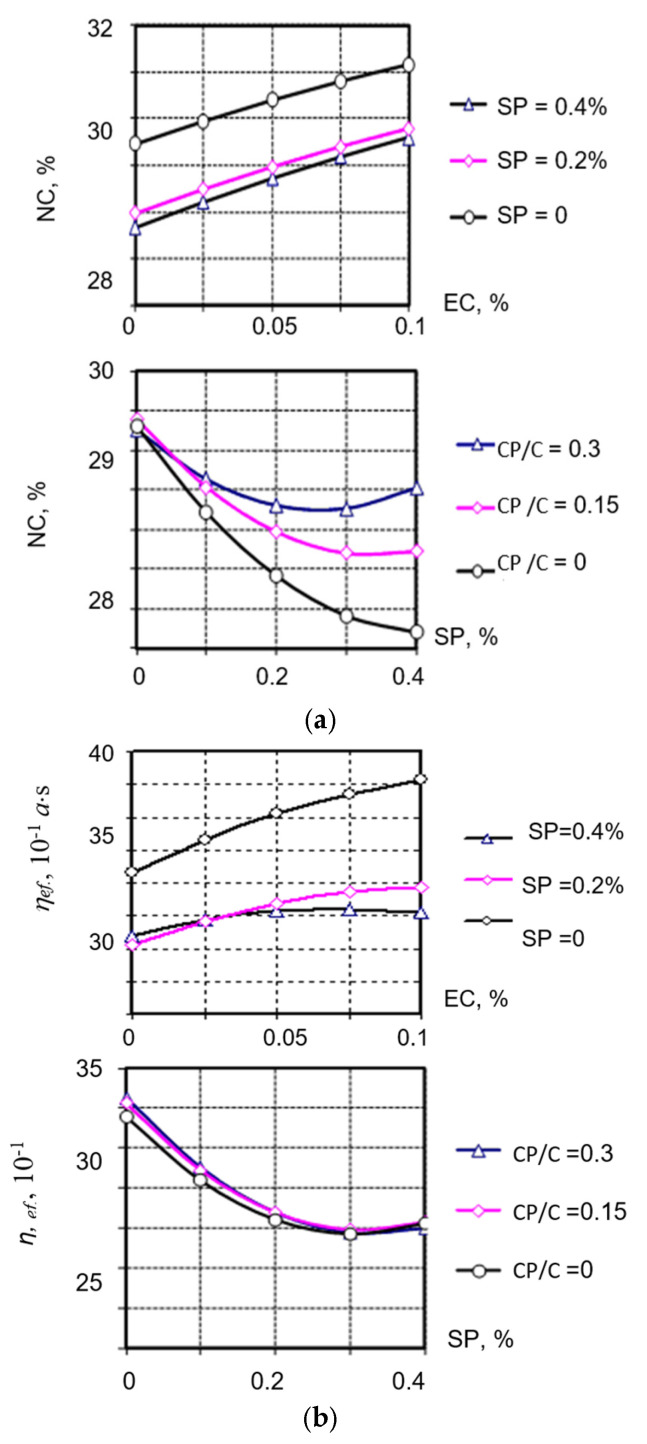
Dependence of the cement pastes’ (**a**) normal consistency and (**b**) effective viscosity on the content of PFM modifier components and the CP/C ratio.

Analysis of the obtained mathematical models (5) and (6) showed that in pastes both without the additive and with CP, the highest decrease in NC and viscosity is achieved when the SP predominates in the PFM composition (Figure 2a,b). At the same time, the highest decrease in viscosity (almost 3 times) is achieved for cement pastes with an SP content equal to 0.4% by the cement weight. For pastes using CP with the same SP content, the viscosity decreases only 1.5 ... 2 times.

Along with normal consistency and viscosity, an important indicator is the hardening kinetics of cement pastes based on CP with the joint action of modifier additives containing SP and EC.

Cement–water dispersions in the process of structure formation and hardening form a group of coagulation–crystallization structures. A characteristic manifestation of the coagulation structure formation and strengthening is cement paste hardening. The effect of surfactants on the cement paste hardening is due to the peculiarities of the adsorption mechanism of their action, increasing the diffusion resistance to the cement grains dissolution, stabilizing the cement–water suspension and, as a result, slowing down the hydration rate. The results of the experiments are presented in Table 7.

Using PFM additives in cement pastes causes a slowdown in the initial setting, which is more significant for pastes with EC. The addition of SP in the range of up to 0.4% by cement weight practically does not change the initial and final setting time, unlike the EC additive, which postpones the initial and final setting time by 30 … 60 min.

### 3.2. Initial Structure Formation of Mortar Mixtures Suitable for 3D Printing

At the second stage of the research, the properties of cement–sand mortar mixtures modified by a complex additive were investigated. One of the main characteristics of the mixture after extrusion from the 3D printer nozzle is its plastic strength, which determines the possibility of applying subsequent mixture layers to the already laid ones.

As is known, the cement systems structure formation process is divided into three stages [9]:

1. Induction period, which occurs immediately after mixing cement with water;
2. Initial or coagulation structure formation, which mainly coincides with setting;
3. Crystallization—hardening and strength gain.

The structural and mechanical characteristic of the initial hardening stages of disperse systems based on binders is plastic strength.

**Figure 3:**
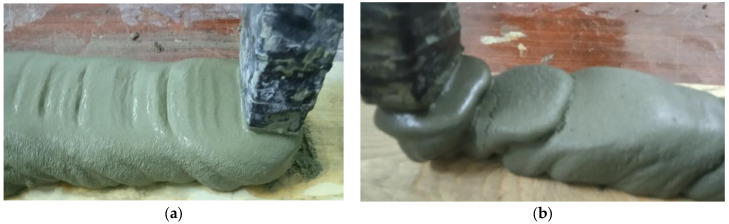
Extrusion of the mixture from a 3D printer: (**a**) satisfactory, (**b**) unsatisfactory with ruptures and influxes.

The mortar mixtures were prepared by mixing Portland cement with the addition of CP and sand in a weight ratio of 1:3 with PFM containing SP, EC and water. The content of CP and additives was according to Table 5. The water content was selected to achieve extrudability from the printer nozzle without breaks and layer formation (Figure 3).

**Figure 4:**
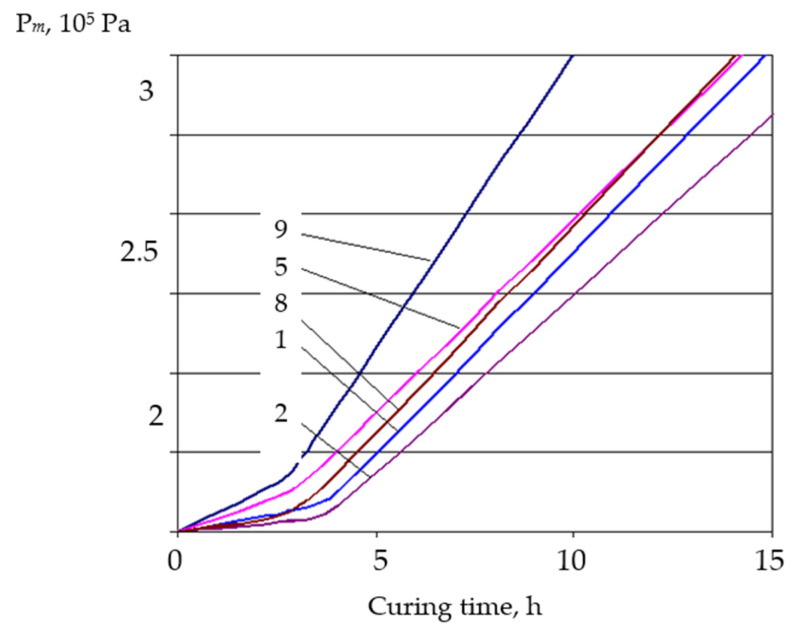
Kinetics in plastic strength change of mixtures with CP additives, in combination with modifiers (composition of cement pastes for preparing the mortar is according to Table 7).

Based on the obtained data, curves of plastic strength (plastograms) were obtained (Figure 4). The quality of the extruded mortar layers was significantly better for mixtures that had high viscosity, which can be estimated by plastic strength. The obtained plastograms have two characteristic areas. The first section of the plastogram approximately corresponds to the hardening time, and the inflection point to the final setting. As is known, plastic strength characterizes the ultimate shear stress of the dispersed system at a certain point of hardening. The change in the cement mortar plastic strength is an integral characteristic of the coagulation and crystallization structure formation processes. At the same time, if the first part of the plastogram characterizes mainly the thixotropic coagulation structure formation, the second corresponds to the coagulation strengthening period and the beginning of crystallization structure formation.

From the obtained plastic strength values, it can be seen that a significant increase begins with a hardening duration exceeding the initial setting. Analysis of plastograms shows that CP and modifier additives, especially with the use of a water-retaining additive, slightly extend the coagulation structure formation period. The addition of SP causes a more intensive growth of plastic strength in the second part of the plastograms.

The second part of the plastograms corresponds to the period of coagulation strengthening and the beginning of the crystallization structure formation. In this part, adding CP in combination with modifier additives causes a significant increase in plastic strength, which agrees with the theoretical premises of their influence on the neoplasm crystallization process.

Already after approximately 2 ... 3 h after the final of setting, the modified mixtures’ plastic strength becomes almost 4 times higher and reaches 2.7 Pa. For unmodified ones, this plastic strength value is achieved after a much longer time period.

With a low water demand of the mixture when using a superplasticizer, and at the same time a sufficient concentration of active filler in the binder–aggregate system, close interparticle contact occurs. It results in forces of various natures, leading to the hardening structure formation. This is evidenced by the increased adhesive strength of mixtures of the specified compositions.

### 3.3. Water Separation, Compressive and Flexural Strength of Cement–Sand Mortars Using CP

To study the influence of the concrete powder content on the water separation and strength of modified mixtures, experiments were carried out following the three-level four-factor B_4_ plan [34]. Since water separation and compressive and flexural strength have a significant dependence on W/C, this indicator was used as one of the influencing factors. In addition, the contents of CP, SP and EC additives were selected as variable factors. The experiment planning conditions are given in Table 8. The water consumption was selected to achieve a cone spread on a shaking table of 200 ... 220 mm, which ensured the necessary extrudability of the mixture from the laboratory printer nozzle. The water separation of mortar was determined after its settling in a cylinder. The experimental data in Table 9 were statistically processed to obtain the regression equations in the coded variables (Table 10).

**Figure 5:**
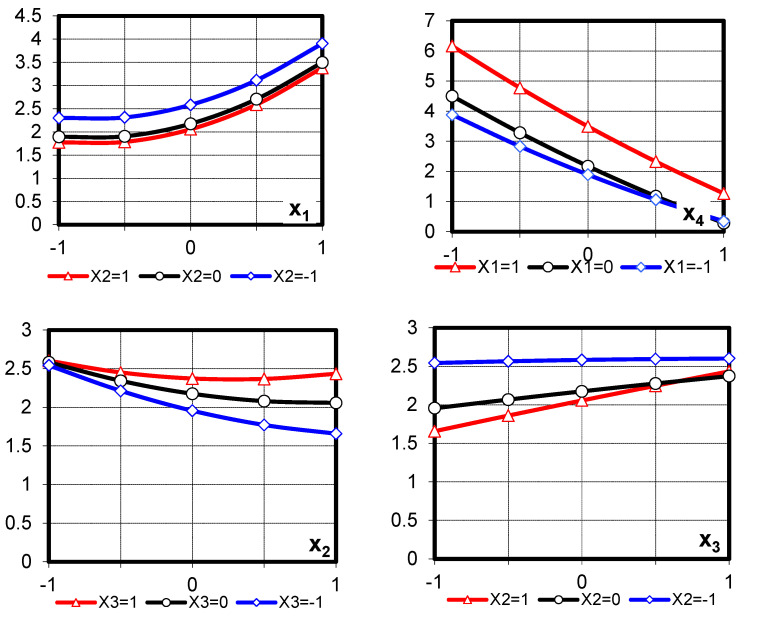
Dependence of mixtures’ water separation on the content of EC and CP/C.

**Figure 6:**
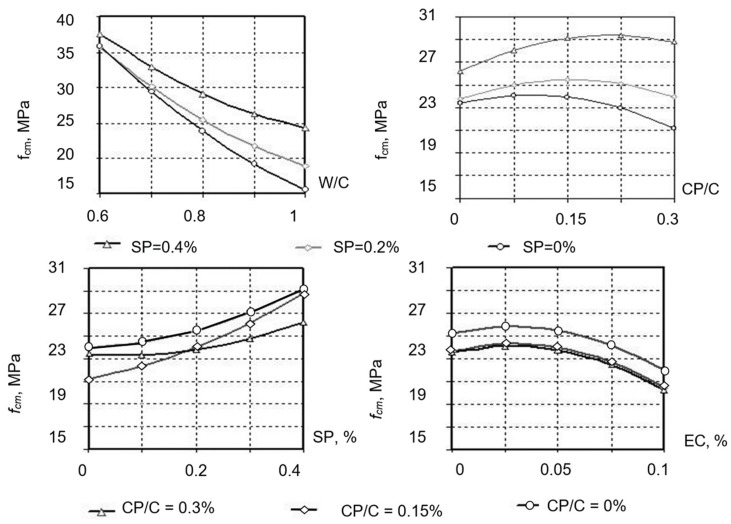
Dependence of the mortars’ compressive strength on composition factors.

**Figure 7:**
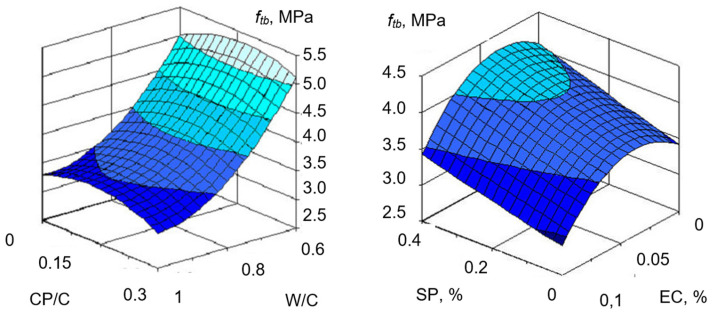
Dependence of the flexural strength of mortars on composition factors.

Based on the obtained data, graphical relationships were obtained to illustrate the influence of composition factors on water separation (Figure 5) and compressive and flexural strength at 28 days (Figure 6 and Figure 7).

The evaluation of the obtained data shows (Figure 5) that, as expected, the addition of CP and increasing its content in the mixture (and, accordingly, water demand), yields higher water separation of mortars. To reduce water separation, it is advisable to use a complex additive containing SP and EC that allows maintaining the NC value of cement paste using CP within the required limits. The water separation of mortar is obviously affected by the ability of cellulose esters to form aquacomplexes that firmly retain water.

The operational reliability of mixtures for a 3D printer, along with their rheological properties, is characterized by the cohesion value, depending on the mortars’ strength indicators, in particular, compressive and flexural strength.

The modified mortars’ strength was investigated using algorithmized experiments. The experiments’ planning conditions are presented in Table 8. Beam specimens that hardened under normal conditions were tested at 28 days. Graphical dependences of compressive strength (*f_cm_*) and flexural strength (*f_tb_*), based on the regression equations (Table 10), are presented in Figure 6 and Figure 7.

Analysis of the obtained results demonstrates that for the investigated cement mortars with CP, W/C has a dominant influence on both compressive and flexural strength. The next most important factor is the SP content. The CP–Portland cement ratio also has an extreme influence on compressive and flexural strength. For all investigated compositions, the CP/C = 0.15 ... 0.2 is close to the optimal one. The strongest influence on the CP–Portland cement ratio is observed for mortars with the maximum SP content and the optimal EC content of 0.03 ... 0.06.

The effect of SP and EC on strength depends on their content. For constant workability, an increase in the SP-3 content in the mixture yields higher strength, which is associated with a decrease in W/C. An increase in the SP content from 0.2 to 0.4% results in the mortar compressive and flexural strengths growing by 23 ... 34% and 18 ... 24%, respectively, with all other factors being equal. An increase in the content of the water-retaining additive leads to a decrease in strength by 10 ... 14%. However, when assessing the effect of these additives, it is worth considering the fact that during extrusion molding and laying the mixture on the previous layer, the negative effect of these additives on the modified mortar strength is offset by the increased mortar workability and high quality of the erected structure. Concrete powder, taking an active part in the hydration and structure formation processes, has a positive effect on the mortars’ strength when SP is used.

Analysis of models (7) and (8) shows that increasing the portion of CP in the binder composition to a certain limit leads to a noticeable increase in the mortar strength at constant values of the water–cement ratio. Increasing the CP content from 10 to 20% increases the compressive and flexural strengths by 13 ... 20% and 9 ... 15%, respectively, depending on SP content. Increasing the CP content above 20% is impractical, since no further increase in strength is observed, and in addition, at a high CP content, the mortar water demand increases noticeably.

The use of a constant-flow SP mixture significantly increases strength by reducing the water–cement ratio, while increasing the water-retaining additive content slightly reduces strength, which is offset by improved extrusion properties and interlayer adhesion. In 3D printing of cement mixtures, interlayer adhesion properties largely depend on the rheology and wettability of the fresh mortar. Reducing the contact angle and increasing water absorption capacity with cellulose ether additives improves material flow on the previous layer and promotes mixture homogenization, which increases the strength of the interlayer bond in 3D concrete. These effects correlate with the general trend toward optimizing the rheological characteristics and the mixture water content for better mutual wetting and sealing of interlayer contacts during filling and structure formation.

The introduction of cellulose ether into the extrusion mixture improves surface wettability by reducing interfacial energy and increasing the hydrophilicity of the system. EC molecules adsorb on the surface of solid particles and form a hydrated polymer shell, facilitating interaction with the aqueous phase and reducing the contact angle. At the same time, the water-holding capacity of the material increases, ensuring the preservation of the surface plasticity of the previous layer and promoting better flow and penetration of the newly extruded material into the microroughness of the substrate. As a result, the actual contact area between layers increases and interlayer adhesion improves [37,38].

## 4. Conclusions

- The study confirms the feasibility and defines the technological conditions for effective use of the powder fraction obtained from crushed concrete waste in 3D printing of building structures.
- The water demand and water separation of mortar are closely related to the cement paste’s normal consistency, which is determined by the mineralogical composition and dispersion of cement, as well as the type and amounts of additives and concrete powder (CP).
- The addition of concrete powder increases the normal consistency of the composite binder, while the use of a superplasticizer (SP) reduces it.
- A combination of SP with a water-retaining additive based on cellulose ethers (EC) allows maintaining the normal consistency at an optimal level, ensuring low water separation and sufficient viscosity of mortars.
- The obtained experimental-statistical models of normal consistency and effective viscosity of cement pastes are adequate at a 95% confidence level and can be used to predict the rheological properties of pastes in a wide range of compositions, including those using CP and various types of polyfunctional modifier (PFM).
- The highest decrease in normal consistency and viscosity is achieved when the SP predominates in the PFM composition.
- The effect of reducing viscosity for pastes with CP is less pronounced than for pastes without it, which indicates an active structure-forming role of CP.
- Using PFM additives slows down the setting time initiation of cement pastes, with cellulose esters having the highest effect.
- The SP in the investigated concentration range practically does not change the setting time, which is important for 3D printing technology.
- CP in combination with modifier additives extends the coagulation structure formation period and provides a more intensive increase in plastic strength at the transition stage to the structure crystallization. Already 2 … 3 h after the final setting, the plastic strength of modified mixtures increases almost 4 times compared to unmodified ones.
- Water separation of mortars increases with an increase in the CP content and water–cement ratio. An effective means of reducing it is the use of the complex SP + EC additive, which is due to the high water-holding capacity of cellulose esters.
- The compression and flexural strength of cement–sand mortars at 28 days is determined primarily by the water–cement ratio, SP content and CP/C ratio. For all investigated compositions, the optimal CP/C ratio is within 0.15 … 0.20.
- Using SP with constant mixture workability contributes to a significant increase in strength by reducing W/C, while increasing the water-retaining additive content slightly reduces strength, which is compensated for by improving extrusion properties and the quality of interlayer joints.
- CP, taking an active part in the hydration and structure formation processes, in combination with rationally selected polyfunctional modifiers, enables the production of mortar with optimal rheological, technological and strength properties suitable for use in 3D printing.

## Figures and Tables

**Table 1:** Mineralogical composition of Portland cement clinker and concrete powder.

Material	C_3_S	C_2_S	C_4_AF	C_3_A	Calcite	Quartz	Feldspars	Hydrated Hardening Products
Clinker, %	57.1	21.27	12.19	6.87	-	-	-	-
CP, %	0.86	2.09	-	-	10.9	42.35	15.5	28.3

**Table 2:** Chemical composition of Portland cement clinker and concrete powder.

Material	Oxide Content, %							
	SiO_2_	Al_2_O_3_	Fe_2_O_3_	CaO	MgO	SO_3_	K_2_O	Na_2_O
Clinker	21.80	5.32	4.11	66.80	0.95	0.63	0.54	0.42
CP	75.5	6.28	3.56	13.2	0.63	-	-	-

The chemical composition of Portland cement based on the used clinker was distinguished by an additional SO_3_ content due to the addition of gypsum in the amount of 3.1%.

**Table 3:** The influence of the CP/C ratio on the cement pastes’ normal consistency.

No.	CP/C	Percentage of PFM (SP + EC) % by the Binder Weight (C + CP)	NC of Cement Pastes, %
1	0	-	26.1
2		0.2 + 0.1	25.7
3		0.2	21.6
4	0.1	-	27.4
5		0.2 + 0.1	26.3
6		0.2	22.7
7	0.2	-	28.1
8		0.2 + 0.1	27.5
9		0.2	22.8
10	0.3	-	28.9
11		0.2 + 0.1	28.1
12		0.2	23.4

**Table 4:** The influence of the CP/CEM ratio on the cement pastes’ normal consistency.

Technological Factors		Level of Variation			Variation Interval
Natural Form	Coded Form	−1	0	1	
Percentage of water-retaining supplement, % of mixture weight, EC	X_1_	0	0.05	0.1	0.05
Percentage of superplasticizer, % by binder weight (C + CP), SP	X_2_	0	0.2	0.4	0.2
CP/C ratio	X_3_	0	0.15	0.3	0.15

**Table 5:** Experimental design matrix and obtained data.

No.	Coded Factor Values			Natural Factor Values			Parameters	
	X_1_	X_2_	X_3_	EC, %	SP, %	CP/C	NC, %	*η_ef_*, 10^−1^ Pa·s
1	1	1	1	0.1	1	0.3	28.5	12.2
2	1	1	−1	0.1	1	0	25.4	11.8
3	1	−1	1	0.1	0	0.3	29.8	33.9
4	1	−1	−1	0.1	0	0	30.4	30.4
5	−1	1	1	0	1	0.3	25.1	7.7
6	−1	1	−1	0	1	0	21.0	9.4
7	−1	−1	1	0	0	0.3	26.8	18.8
8	−1	−1	−1	0	0	0	26.5	17.4
9	1	0	0	0.1	0.2	0.15	27.5	19.3
10	−1	0	0	0	0.2	0.15	23.9	10.6
11	0	1	0	0.05	1	0.15	25.4	15.7
12	0	−1	0	0.05	0	0.15	28.8	30.5
13	0	0	1	0.05	0.2	0.3	26.6	13.9
14	0	0	−1	0.05	0.2	0	24.8	13.0
15	0	0	0	0.05	0.2	0.15	25.9	16.9
16	0	0	0	0.05	0.2	0.15	28.5	12.2
17	0	0	0	0.05	0.2	0.15	25.4	11.8

**Table 6:** Experimental and statistical models of NC and *η_ef_* of cement pastes with CP and PFM additives.

Pastes Properties	Regression Equation	
NC, %	25.9 + 1.82X_1_ − 1.67X_2_ + 0.89X_3_ − 0.172X_1_^2^ + 1.18X_2_^2^ − 0.222X_3_^2^ + 0.11X_1_X_2_ − 0.24X_1_X_3_ + 0.94X_2_X_3_	(5)
*η_ef_*, 10^−1^ Pa·s	16.9 + 4.37X_1_ − 7.44X_2_ + 0.45X_3_ − 1.95X_1_^2^ + 6.20X_2_^2^ − 3.45X_3_^2^ − 2.65X_1_X_2_ + 0.525X_1_X_3_ − 0.75X_2_X_3_	(6)

**Table 7:** Setting times of cement pastes using CP.

No.	CP/C	PFM, %		Setting Time, h-min	
		SP	EC	Initial	Final
1	-	-	-	1–30	3–30
2	0.15	-	-	1–35	3–35
3	0.3	-	-	1–40	3–40
4	0.15	0.2	-	1–45	3–40
5	0.15	0.2	0.05	2–25	4–35
6	0.3	0.2	-	1–50	3–45
7	0.3	0.2	0.05	2–30	4–40
8	0.3	0.2	0.1	2–50	4–50
9	0.15	0.4	0.05	2–35	4–45
10	0.3	0.4	0.05	2–40	4–50

**Table 8:** Experiment planning conditions.

Factors		Variation Levels			Variation Interval
Natural Form	Coded Form	**−1**	**0**	**1**	
W/C	X_1_	0.6	0.8	1.0	0.2
CP/C	X_3_	0	0.15	0.3	0.15
SP, % of (C + CP) weight	X_3_	0	0.2	0.4	0.2
Water-retaining supplement percentage, % of mixture weight, EC	X_4_	0	0.05	0.1	0.05

**Table 9:** Experimental results.

Point No.	Coded Factor Values				Natural Factor Values				Water Separation, %	Strength at 28 Days, MPa	
	X_1_	X_2_	X_3_	X_4_	W/C	CP/C	SP, %	EC, %		Flexural	Compressive
1	1	1	1	1	1.0	0.3	0.4	0.1	1.60	2.6	21.7
2	1	1	1	−1	1.0	0.3	0.4	0	6.85	3.3	25.3
3	1	1	−1	1	1.0	0.3	0	0.1	1.40	2.0	10.9
4	1	1	−1	−1	1.0	0.3	0	0	4.50	2.6	13.8
5	1	−1	1	1	1.0	0	0.4	0.1	1.80	2.7	16.4
6	1	−1	1	−1	1.0	0	0.4	0	6.80	3.5	20.0
7	1	−1	−1	1	1.0	0	0	0.1	0.71	2.2	10.4
8	1	−1	−1	−1	1.0	0	0	0	7.10	2.8	13.3
9	−1	1	1	1	0.6	0.3	0.4	0.1	0.60	4.6	34.3
10	−1	1	1	−1	0.6	0.3	0.4	0	3.85	5.2	37.9
11	−1	1	−1	1	0.6	0.3	0	0.1	0.24	4.1	30.6
12	−1	1	−1	−1	0.6	0.3	0	0	3.50	4.5	33.5
13	−1	−1	1	1	0.6	0	0.4	0.1	0.52	4.5	30.3
14	−1	−1	1	−1	0.6	0	0.4	0	4.42	5.1	33.9
15	−1	−1	−1	1	0.6	0	0	0.1	0.71	4.0	31.4
16	−1	−1	−1	−1	0.6	0	0	0	4.62	4.4	34.3
17	1	0	0	0	1.0	0.15	0.2	0.05	3.70	3.5	18.9
18	−1	0	0	0	0.6	0.15	0.2	0.05	1.70	5.3	35.7
19	0	1	0	0	0.8	0.3	0.2	0.05	2.05	3.6	25.0
20	0	−1	0	0	0.8	0	0.2	0.05	2.60	3.7	22.8
21	0	0	1	0	0.8	0.15	0.4	0.05	2.20	4.2	29.1
22	0	0	−1	0	0.8	0.15	0	0.05	2.14	3.6	23.9
23	0	0	0	1	0.8	0.15	0.2	0.1	0.61	3.2	22.0
24	0	0	0	−1	0.8	0.15	0.2	0	4.19	3.7	25.3

**Table 10:** Experimental-statistical models.

Property	Model	
Water separation, %	W*_sep_* = 2.18 + 0.8X_1_ − 0.26X_2_ + 0.21X_3_ − 2.11X_4_ + 0.52X_1_^2^ + 0.14X_2_^2^ − 0.01X_3_^2^ − 0.22X_4_^2^ + 0.001X_1_X_2_ + 0.189X_1_X_3_ − 0.338X_1_X_4_ + 0.179X_2_X_3_ + 0.272X_2_X_4_ − 0.047X_3_X_4_	(7)
Flexural strength at 28 days, MPa	*f_tb_* = 3.93 − 0.935X_1_ − 0.017X_2_ + 0.291X_3_ − 0.289X_4_ + 0.459X_1_^2^ − 0.267X_2_^2^ + 0.009X_3_^2^ − 0.491X_4_^2^ − 0.069X_1_X_2_ − 0.044X_1_X_4_ − 0.031X_3_X_4_	(8)
Compressive strength at 28 days, MPa	*f_cm_* = 25.5 − 8.4X_1_ + 1.11X_2_ + 2.61X_3_ − 1.62X_4_ + 1.83X_1_^2^ − 1.62X_2_^2^ + 1.03X_3_^2^ − 1.87X_4_^2^ + 0.32X_1_X_2_ + 1.77X_1_X_3_ + 1.21X_2_X_3_ − 0.18X_3_X_4_	(9)

## Data Availability

The original contributions presented in this study are included in the article. Further inquiries can be directed to the corresponding author.
